# Open Access Meets Discoverability: Citations to Articles Posted to Academia.edu

**DOI:** 10.1371/journal.pone.0148257

**Published:** 2016-02-17

**Authors:** Yuri Niyazov, Carl Vogel, Richard Price, Ben Lund, David Judd, Adnan Akil, Michael Mortonson, Josh Schwartzman, Max Shron

**Affiliations:** 1 Academia.edu, San Francisco, California, United States of America; 2 Polynumeral, New York, New York, United States of America; Universidad de Las Palmas de Gran Canaria, SPAIN

## Abstract

Using matching and regression analyses, we measure the difference in citations between articles posted to Academia.edu and other articles from similar journals, controlling for field, impact factor, and other variables. Based on a sample size of 31,216 papers, we find that a paper in a median impact factor journal uploaded to Academia.edu receives 16% more citations after one year than a similar article not available online, 51% more citations after three years, and 69% after five years. We also found that articles also posted to Academia.edu had 58% more citations than articles only posted to other online venues, such as personal and departmental home pages, after five years.

## Introduction

Academia.edu is a website where researchers can post their articles and discover and read articles posted by others. It combines the archival role of repositories like ArXiv, SSRN, or PubMed with social networking features, such as profiles, news feeds, recommendations, and the ability to follow individuals and topics. The site launched in 2008 and as of January 2016 has approximately 30 million registered users who have uploaded approximately 8.5 million articles. Registration on the site is free and users can freely download all papers posted to the site.

There is a large body of research on the citation advantage of open access articles, and researchers are still debating the size and causes of the advantage. Some studies have found that open access articles receive substantially more citations than pay-for-access articles, even after controlling for characteristics of the articles and their authors [1, 2]. Other studies using experimental and quasi-experimental methods have concluded that any measured citation advantage is mostly due to selection bias and other unobserved differences between free and paid articles [3–5].

Both the supportive and critical studies have focused on the accessibility of articles: once found, can the article be obtained for free? They have given less consideration to the discoverability of articles: how easily can the article be found? This makes sense; the methods researchers often use to find articles don’t privilege open access over paid sources or vice versa. Google Scholar, for example, returns both free and paid sources, as do many library databases.

Academia.edu, on the other hand, has unique features for discovering articles, making it an interesting venue for analyzing a citation advantage. Users are notified when authors they follow post articles to the site. They can then share those articles with their followers. A user can tag an article with a subject like “High Energy Physics” and users following that subject will be notified about the paper.

A number of users have reported to the Academia.edu team that they observed increased citations after posting their articles to the site [6, 7]. Motivated by those anecdotal reports, a formal statistical analysis was conducted of the citation advantage associated with posting an article.

We find that a typical article posted on Academia.edu receives approximately 16% more citations compared to similar articles not available online in the first year after upload, rising to 51% after three years, and 69% after five years. We also find that a typical article posted on Academia.edu receives more citations than an article available online on a non-Academia.edu venue, such as a personal homepage, a departmental homepage, or a journal site. A typical paper posted only to Academia.edu receives 15% *fewer* citations than an article uploaded to a non-Academia.edu site in the first year, but 19% *more* after three years, and 35% after five years.

Our study is observational, requiring us to carefully account for possible sources of selection bias. We find that the citation advantage persists even after controlling for a number of possible selection biases.

## Background

### The Open Access Citation Advantage

Even though Academia.edu differs from traditional venues for open access, the hypotheses and methods in this paper overlap with research on the open access citation advantage. The term “open access” typically refers to articles made freely available according to specific Open Access policies of academic journals: for example “Gold Open Access” policies where authors or institutions pay the journal to make an article freely available, or “Green Open Access” where an author may archive a free version their article online. Sometimes, though, “open access” is used more loosely to refer to any manner by which articles are made freely available online. Some authors use the term “free access” for this broader definition, to distinguish it from Green and Gold Open Access policies. Our study does not rely on these distinctions, and we will use the terms “open access” and “free access” interchangeably to refer to the broader definition of freely downloadable articles.

Many researchers, beginning with [8], have found that free-access articles tend to have more citations than pay-for-access articles. This citation advantage has been observed in a number of studies, spanning a variety of academic fields including computer science [8], physics [9], and biology and chemistry [1].

The estimated size of the citation advantage varies across and even within studies, but is often measured to be between 50% and 200% more citations for open access articles.[10] The variety of estimates is unsurprising, since both open access and citation practices vary widely across disciplines, and citations accumulate at different rates for different articles published in different venues. Different statistical methods also lead to different estimates. Some studies have simply compared unconditional means of citations for samples of free and paid articles [8], while others, such as [1] measured the advantage in a regression analysis with a battery of controls for characteristics of the articles and their authors.

### Critiques of the Citation Advantage

Other studies have presented evidence against an open access citation advantage, arguing that although there is correlation between open access and more citations, open access does not cause more citations. (See, e.g., [11] and [12] for critical reviews of the citation advantage literature.)

Kurtz et al. [13]—in a framework adopted by several subsequent authors (e.g., [3, 11, 14].)—put forth three postulates to explain the correlation between open access and increased citations:

1. **The Open Access postulate.** Since open access articles are easier to obtain, they are easier to read and cite.
2. **The Early View postulate.** Open access articles tend to be available online prior to their publication. They can therefore begin accumulating citations earlier than paid-access articles published at the same time. When comparing citations at fixed times since publication, the open-access articles will have more citations, because they have been available for longer.
3. **The Selection Bias postulate.** If more prominent authors are more likely to provide open access to their articles, or if authors are more likely to provide access to their “highest quality” articles, then open access articles will have more citations than paid-access articles.

Kurtz et al. [13], and later [14], concluded that the Early View and Selection Bias effects were the main drivers of the correlation between open-access and increased citations. A lack of causal open-access effect was further supported in other studies, such as the randomized trials in [3] and [4], and the instrumental variables regressions in [5].

But even these studies are not conclusive. For example, Kurtz et al. [13] point out that their conclusions may be specific to their sample: articles published in the top few astronomy journals. The experimental treatment in [3] and [4] was to make randomly-chosen articles free to download on the publisher’s website. How easily researchers could determine these articles were available for free is unclear. And, while the instrumental variable analysis of [5] found evidence of selection bias in open access, they still estimated a statistically and practically significant citation advantage even after controlling for that bias.

Regardless of the validity or generality of their conclusions, these studies do establish that any citation advantage analysis must take into account the effects of time and selection bias on citation differentials.

### Sources of Selection Bias in Academia.edu Citations

Like most citation advantage studies, ours is observational, not experimental. Articles are not uploaded to Academia.edu randomly. Authors choose to register as users on the site, and then choose which of their articles to upload. When making comparisons to articles not posted to the site, this creates several potential sources of bias in unconditional citation comparisons.

1. **Self-selection of disciplines.** Academia.edu users may be more likely to come from particular disciplines. Since the citation frequency differs across disciplines, a citation advantage estimate that doesn’t control for academic discipline might over- or underestimate the true advantage.
2. **Self-selection of authors.** Researchers who post papers on Academia.edu might differ from those who do not. Users might skew younger, or be more likely to work at lesser-known institutions. If so, we would expect to find that papers posted to the site tend to have fewer citations than those not. Or users might skew in the other direction—having more established reputations, or coming from better-known institutions, in which case we could overestimate the actual advantage. Furthermore, users who post papers may also be generally more proactive about distributing and marketing their work, both through Academia.edu and other venues online and off. If this were true, it would also cause us to overestimate the actual advantage.
3. **Self-selection by article quality.** Even if Academia.edu users were not systematically different than non-users, there might be systematic differences between the papers they choose to post and those they do not. As [13] and others have hypothesized, users may be more likely to post their most promising, “highest quality” articles to the site, and not post articles they believe will be of more limited interest.
4. **Self-selection by type of article.** Academic journals publish content besides original research or scholarship: book reviews, errata, responses to recently published articles, conference abstracts, editorials, etc. These other types of content typically receive fewer citations than research articles. If Academia.edu users are less likely to post these other types of content to the site, then we might overestimate the advantage relative to an off-Academia group that contains more “non-research” content.
5. **Self-selection by article availability.** A user may be more likely to post a paper to the site if they have already made it available through other venues, such as their personal website or institutional or subject-specific repositories. In this case, a citation advantage estimated for Academia.edu papers might be measuring in part or whole, a general open access effect from the articles’ availability at these other venues.

Many of these factors cannot be observed directly or completely, and their aggregate effect on citation advantage estimates is difficult to predict. We have collected data and employed matching and regression strategies to mitigate each of the above potential biases, and continue to find a substantive citation advantage to articles posted to Academia.edu.

## Materials and Methods

We rely on data from several sources: (1) articles the Academia.edu website, (2) citation counts and free-access status from Google Scholar, (3) journal rankings from SCIMago/Scopus, and (4) journal research fields from the Australian Research Council. All data and code used in the analysis are available for download at https://github.com/polynumeral/academia-citations.

### On-Academia and Off-Academia Articles

Our analysis is a comparison of citations between articles posted to Academia.edu to articles not posted. We refer to these two samples as the “On-Academia” sample and the “Off-Academia” sample. Articles comprising each sample were selected in the following way.

**On-Academia Sample:** The articles in our analysis were uploaded to the Academia.edu between 2009 and 2012, inclusive. We chose to start at 2009 because this was the first full year that the site was active. We stopped at 2012 so that all articles in the sample are at least two-years old and have had time to accumulate citations. We restrict our sample to articles that were posted to the site in the same year they were published. We refer to this as the “P = U” (Published = Uploaded) restriction. This ensures that all of the articles are exposed to any citation advantage effect starting from their publication. It also mitigates bias from authors favoring their, *ex post*, most-cited articles when uploading to the site.

Our analysis relies on information from Google Scholar and CrossRef. The latter is a database containing journals, articles, authors, and Digital Object Identifiers (DOIs). Therefore, we restricted the on-Academia sample to articles that could be matched by title and author to both Google Scholar results and CrossRef entries.

**Off-Academia Sample:** Using the CrossRef database, we selected a random subset of articles published in the years as articles in the on-Academia sample, but which had not been posted to Academia.edu.

### Citation Counts

For all articles in both the on- and off-Academia samples, we obtained citation counts from Google Scholar between April and August 2014.

Table 1 shows the number of articles in each cohort and sample. The on-Academia sample each year is a subset of papers posted to the site that year. We excluded papers uploaded to the site that were published in an earlier year, and papers that could not be matched to a Google Scholar search result or a CrossRef entry based on their titles and authors. Users manually enter a paper’s title when they upload it to the site, and what they enter may differ from the paper’s canonical title. (For example, a user may add “forthcoming in PLoS” to the title.) This sort of discrepancy was a common reason for a failure to match. We do not believe that failure to match a paper is related to its citations, and therefore these exclusions should not bias our results.

**Table 1:** Sample size of papers, by cohort.

Year	Off-Academia	On-Academia
2009	4,600	149
2010	5,768	490
2011	6,989	2,236
2012	8,368	2,616
**Total**	25,725	5,491

Articles in the sample come from 5,725 different journals, but there is a concentrated representation of journals. Table 2 lists the ten journals with the highest number of articles in our sample. The most-represented journal, Analytical Chemistry comprises 4.6% of the sample, and the top ten journals comprise 12%.

**Table 2:** Journals with the most number of articles in the sample.

Journal	# Articles	% Total
Analytical Chemistry	1,422	4.56%
Biological and Pharmaceutical Bulletin	329	1.05%
Analytical Methods: advancing methods and applications	316	1.01%
Analytical Biochemistry	303	0.97%
Bioconjugate Chemistry	285	0.91%
Applied Mechanics and Materials	282	0.90%
PLoS One	194	0.62%
Applied Physics Letters	179	0.57%
AAPS PharmSciTech	164	0.53%
Anesthesia and Analgesia	155	0.50%

### Journal Impact Factors and Divisions

We used the impact factor of an article’s journal as a matching variable and regression predictor. Journal impact factors were obtained from SCIMago Journal and Country Rank, which uses citation data from Scopus [15]. The metric we refer to as the “impact factor” is the “Cites per Doc, 2 year” metric on the SCIMago site. A journal’s impact factor in, for example 2012, is calculated as the average number of citations received in 2012 by papers that were published in the journal in 2010 and 2011. We matched each article to its journal’s impact factor in the year the article was published. This ensures that the impact factor was not affected by the article itself, only articles published in the journal in prior years. The journals in our sample with the highest impact factors are listed in Table 3.

**Table 3:** Top ten journals in sample by impact factor. Impact factor is averaged by year.

Journal	Impact Factor
Chemical Reviews	41.92
Annual Review of Immunology	39.88
Chemical Society Reviews	31.76
Annual Review of Biochemistry	31.52
Annual Review of Astronomy and Astrophysics	28.48
Nature Reviews Neuroscience	28.34
Nature Materials	28.26
Progress in Polymer Science	26.7
Nature	25.87
Lancet Oncology	25.48

We also obtained data on the journals’ fields of research from the Australian Research Council’s *Excellence in Research for Australia* report [16]. The report contains data on academic journals that includes labels for their Fields of Research, defined using a hierarchical taxonomy from the Australian New Zealand Standard Research Classification [17]. Field of Research is the second level of taxonomy, and the journals in our sample cover around 200 different Fields.

We instead rely on the first level of the taxonomy, the “Division” of the journal, which describes broad disciplines of research. There are 22 Divisions in the taxonomy and a journal can be labelled with up to three different Divisions. Multidisciplinary journals, which cover more than three Fields of Research, are labelled with a 23rd Division label of “Multidisciplinary.”

All of the analyses in the paper were also conducted with the “Field of Research” labels, using text analysis and dimension reduction techniques to account for the large number of labels and high correlations amongst them. These analyses gave nearly identical results to those based on the Division labels, so we use the latter since they are easier to interpret.

Table 4 provides summary data about the Divisions in our sample: the share of articles in the full and on- and off-Academia samples in each discipline, and the median impact factor of journals in our sample in each Division. Nearly a third of articles in our sample are in Medical and Health Sciences journals, while Engineering and Biological Sciences each represent a fifth of articles. The columns add up to more than 100% because journals can be labeled with up to three disciplines.

**Table 4:** Journal Divisions, defined according to the taxonomy in [17]. Share of articles in the full sample, the on-Academia sample, and the off-Academia sample in each Division, and the median impact factor of sample articles in the Division. Journals can be labelled with between one and three disciplines.

Division	% All	% On	% Off	Med. IF
Medical and Health Sciences	33.0%	18.6%	36.1%	2.58
Engineering	22.9%	12.0%	25.3%	2.77
Biological Sciences	20.6%	19.6%	20.8%	2.55
Chemical Sciences	18.7%	6.3%	21.4%	3.79
Psychology and Cognitive Sciences	7.7%	17.5%	5.6%	2.46
Physical Sciences	7.2%	8.3%	7.0%	2.41
Mathematical Sciences	7.1%	5.0%	7.5%	1.36
Multidisciplinary	5.1%	11.5%	3.7%	3.20
Information and Computing Sciences	4.9%	5.2%	4.8%	1.95
Earth Sciences	4.0%	8.7%	2.9%	2.28
Studies in Human Society	3.7%	9.8%	2.4%	1.15
Agricultural and Veterinary Sciences	3.7%	4.6%	3.5%	2.16
Environmental Sciences	3.4%	5.3%	3.0%	2.48
Commerce, Management, Tourism and Services	2.8%	4.4%	2.5%	1.30
Technology	2.2%	1.9%	2.3%	1.96
Education	1.8%	4.5%	1.2%	1.12
Economics	1.6%	1.9%	1.5%	1.15
Language, Communication and Culture	1.4%	4.6%	0.8%	0.63
Philosophy and Religious Studies	1.4%	4.2%	0.8%	0.64
History and Archaeology	1.3%	4.8%	0.5%	0.92
Built Environment and Design	0.9%	1.7%	0.8%	1.84
Creative Arts and Writing	0.5%	1.4%	0.3%	0.76
Law and Legal Studies	0.4%	0.8%	0.3%	0.77

### Document Types

We include in our analysis only articles with original research, analysis or scholarship, or survey articles. We exclude book reviews, editorials, errata, and other “non-research” content. Our procedure for obtaining on- and off-Academia articles provided 37,266 articles. From this sample, we removed any articles not identified to be original research.

To identify the type of each article, we used Amazon Mechanical Turk (MTurk), a crowd-sourcing marketplace. Common uses of MTurk in academic research include collecting survey data, performing online experiments, and classifying data to train and validate machine learning algorithms. An appendix with a more complete description of the document classification process, including the worker questionnaire, and accuracy statistics, is available at this paper’s Github repo, https://github.com/polynumeral/academia-citations/. The repo also includes underlying data on worker responses.

We provided DOI links to articles in our sample to over 300 MTurk workers. The workers were asked to fill out an online form based on information from the abstract or full text at the DOI link. They were finally asked to classify the article as one of the following types:

1. A summary of a meeting or conference
2. An Editorial or Commentary
3. A response to a recent article in the same journal;
4. An article with original research, analysis or scholarship, or a broad survey of research on a topic
5. This is a Book Review, Software Review, or review of some other recent work or performance
6. An Erratum, Correction, or Retraction of an earlier article
7. Something else

Workers might fail to categorize an article, giving one of these reasons: the link was broken, there was no abstract or text available on the site, the article was in a foreign language, or they otherwise couldn’t tell. Some workers’ results were excluded if they exhibited suspicious patterns, such as giving all articles the same classification, or completing a large number of tasks in an unreasonably short time. Their tasks were then resubmitted so that each article had three independent reviews.

Each article was reviewed by three different workers. Our sample only includes articles that all three workers identified as “original research” (option 4). Of the original 37,266 articles, this left 31,216 “original research” articles. Relying on a majority, 2-of-3 vote to classify articles would have resulted in 35,311 “original research” articles. Unanimity is a conservative classification rule, but given that false positive classification of “original research” articles could upwardly bias our result, we consider it appropriate.

### Online Availability

In the last section, we considered several potential sources of selection bias in the on-Academia sample. One was that users might be more likely to upload articles to the site if they have also made those articles available elsewhere online. To examine this possibility, we collected data on whether all papers in our sample were freely available from non-Academia sources. For the on-Academia articles, this would mean they were available from at least two online sources.

To determine whether a paper was available elsewhere, we searched for its title on Google Scholar, and checked whether the results contained a link to a non-paywalled full-text article. This method is subject to false negatives, but since the failure to match a title, or correctly identify a full-text article on a non-Academia site should be independent of whether the article is also posted to Academia, we expect its error rate to be similar for both on- and off-Academia articles.

Table 5 lists the number of articles searched, and the percentage with free-access to full text on non-Academia.edu sites. We find that papers in the on-Academia.edu sample are more likely to be available online as papers in the off-Academia sample. This indicates that there may be some self-selection by availability in our data. Our regression analyses control for online-availability, mitigating potential bias from the discrepancy.

**Table 5:** Share of sample articles freely available from non-Academia.edu sites.

		Off-Academia	On-Academia
a.	Full-text available elsewhere	9,487	3,652
b.	Articles searched	25,725	5,491
c.	Share *(a ÷ b)*	36.9%	66.5%

The use of a binary indicator for online availability does conceal some potentially useful information about the article’s availability. For example, how many different venues is may be available on, or what those specific venues are. Such metrics are difficult to measure accurately, but could be interesting. Indeed, this paper argues that venue-specific effects can be meaningful. Nonetheless, we do not believe this un-measured information will contribute to any substantial bias for several reasons; the primary one being that we find a significant citation advantage amongst articles that are *not* online on any non-Academia venue; an effect generally larger than the average online advantage we measure with the binary variable. Were we to use a richer metric for online availability, those article would not be affected, and their Academia advantage would remain roughly the same.

### Quantifying the Citation Advantage

Our general empirical strategy is to estimate the distribution of the citation count of article *i*, published in journal *j* at time *t*, conditional on it being posted to Academia.edu, and compare this distribution to the same article, but conditional on it not being posted to the site. Denoting the number of citations as a random variable *Y*, we are interested in the distributions

$$\begin{array}{cc} P_{ijt}^{1}\left(y\right) & =\text{Prob}\left(Y\leq y\;|\;j,t,\text{on-Academia}\right)\\ P_{ijt}^{0}\left(y\right) & =\text{Prob}\left(Y\leq y\;|\;j,t,\text{off-Academia}\right). \end{array}$$

We can compute the change in an article’s citations associated with posting to Academia.edu, Δ_*ijt*_, by comparing summary statistics of these distributions. For example, the difference in means

$$\begin{array}{c} \Delta_{ijt}=E_{ijt}^{1}\left(Y\right)-E_{ijt}^{0}\left(Y\right), \end{array}$$

or medians,

$$\begin{array}{c} \Delta_{ijt}={\text{Med}}_{ijt}^{1}\left(Y\right)-{\text{Med}}_{ijt}^{0}\left(Y\right). \end{array}$$

One approach would be to directly estimate these summary statistics by computing average or median citations within each journal × year group. Unfortunately many of these groups contain too few articles to accurately estimate summary statistics. Instead, we use journal-specific covariates to represent journals, most prominently the journal’s impact factor. This leads to two approaches: a non-parametric matching analysis, and a regression analysis.

### Properties of Citation Count Distributions

**Fig 1:**
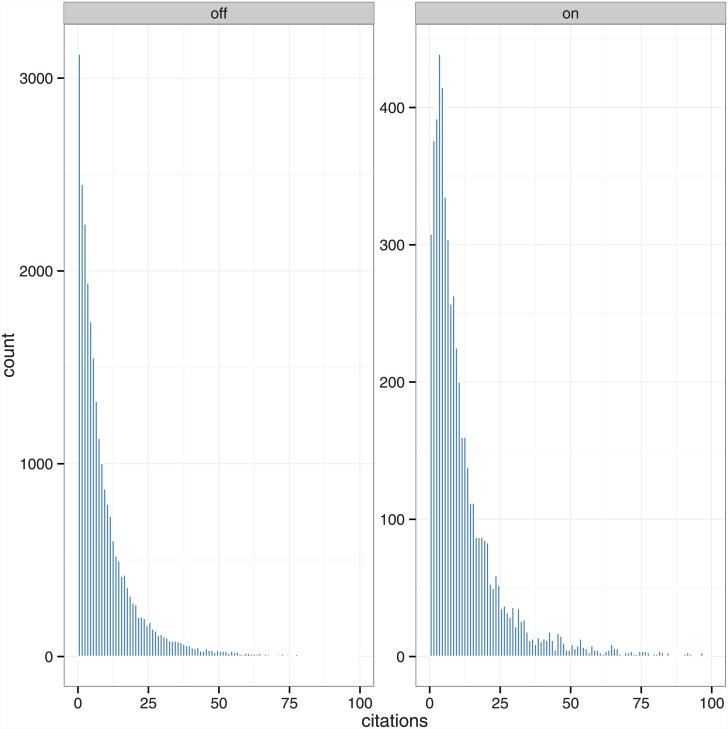
Distributions of citations (x-axis is truncated at 100).

Citation counts are non-negative integers with a highly right-skewed distribution. This can be seen in Table 6 and Fig 1, the latter of which also shows that the modal article has one or no citations. Our matching analysis accounts for this aspect of the data by comparing quantiles of on- and off-Academia citation counts. Our regression analysis applies several parametric models that accommodate right-skewed count data.

**Table 6:** Citations summary statistics.

Sample	Min.	1st Qu.	Median	Mean	3rd Qu.	Max.
off-Academia	0	2	5	10.19	12	1237
on-Academia	0	3	7	12.77	15	721

## Results

### Matching by Impact Factor

Our first analysis compares citations of on- and off-Academia articles grouped by cohort and their journals’ impact factors. This is effectively a matching strategy with year and impact-factor as the covariates; the purpose being to provide a relatively simple non-parametric estimator of the difference while controlling for important covariates. The regression analyses in the subsequent sections will expand on this analysis with a larger array of controls.

To match on-Academia articles to off-Academia articles, we computed decile bins of impact factors amongst the on-Academia articles in a cohort. Therefore, each impact factor bin represents 10% of articles in the on-Academia sample for that year. We then grouped the off-Academia articles into those bins, and compared samples within each bin.

**Fig 2:**
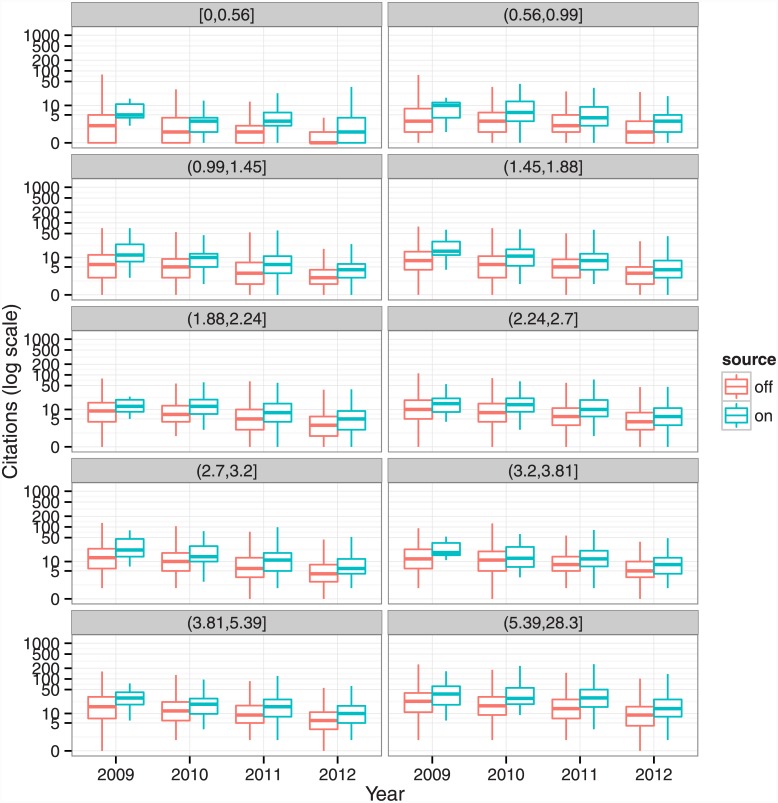
Boxplots of off- and on-Academia article citations, by cohort and impact factor bin.

Fig 2 shows boxplots of citations to on- and off-Academia articles in each cohort and impact factor bin. (Bornmann et al. [18], among others, advocate using boxplots to compare citation differences across samples.) Evident in the figure are that older papers have more citations, and that articles published in higher impact factor journals have more citations. Furthermore, we find that median number of citations to on-Academia articles is consistently higher than off-Academia articles across cohorts and impact factor bins. Table 7 provides the medians and citation advantages for each of the comparison groups. The on-Academia citation advantage ranges from 1 extra citation for low impact factor bins to 15 for high impact bins. For low impact factor bins, the advantage is large in percentage terms—2 or 3 extra citations is a 200% increase.

**Table 7:** Median citations by cohort and impact factor bin for off- and on-Academia.edu samples.

Year	Impact Factor Bin	Off-Academia	On-Academia	Abs. Diff	% Diff.
2009	[0,0.56]	2	5.0	3.0	150
	(0.56,0.99]	3	10.0	7.0	233
	(0.99,1.45]	6	12.0	6.0	100
	(1.45,1.88]	8	15.5	7.5	94
	(1.88,2.24]	9	12.5	3.5	39
	(2.24,2.7]	10	15.0	5.0	50
	(2.7,3.2]	13	22.0	9.0	69
	(3.2,3.81]	12	18.5	6.5	54
	(3.81,5.39]	16	29.5	13.5	84
	(5.39,28.3]	23	37.5	14.5	63
2010	[0,0.56]	1	3.0	2.0	200
	(0.56,0.99]	3	6.0	3.0	100
	(0.99,1.45]	5	10.0	5.0	100
	(1.45,1.88]	6	11.0	5.0	83
	(1.88,2.24]	7	12.5	5.5	79
	(2.24,2.7]	8	14.0	6.0	75
	(2.7,3.2]	10	14.0	4.0	40
	(3.2,3.81]	11	12.5	1.5	14
	(3.81,5.39]	12	19.0	7.0	58
	(5.39,28.3]	17	28.0	11.0	65
2011	[0,0.56]	1	3.0	2.0	200
	(0.56,0.99]	2	4.0	2.0	100
	(0.99,1.45]	3	6.0	3.0	100
	(1.45,1.88]	5	8.0	3.0	60
	(1.88,2.24]	5	8.0	3.0	60
	(2.24,2.7]	6	10.0	4.0	67
	(2.7,3.2]	6	11.0	5.0	83
	(3.2,3.81]	8	12.0	4.0	50
	(3.81,5.39]	9	16.0	7.0	78
	(5.39,28.3]	14	29.0	15.0	107
2012	[0,0.56]	0	1.0	1.0	—
	(0.56,0.99]	1	3.0	2.0	200
	(0.99,1.45]	2	4.0	2.0	100
	(1.45,1.88]	3	4.0	1.0	33
	(1.88,2.24]	3	5.0	2.0	67
	(2.24,2.7]	4	6.0	2.0	50
	(2.7,3.2]	4	6.0	2.0	50
	(3.2,3.81]	5	8.0	3.0	60
	(3.81,5.39]	6	10.0	4.0	67
	(5.39,28.3]	9	14.0	5.0	56

**Fig 3:**
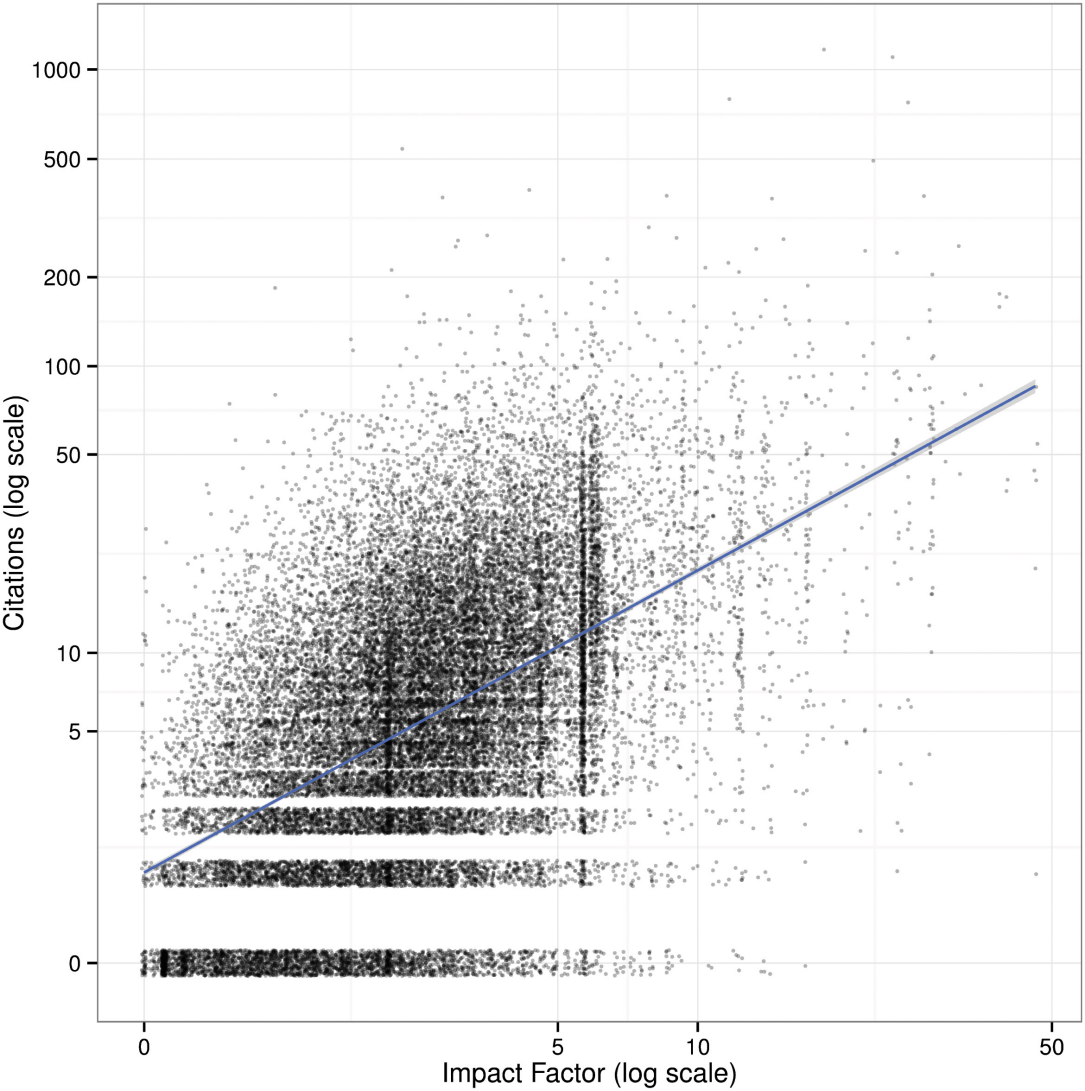
Article citations against Impact Factor (log scale).

Using impact factors to match on- and off-Academia articles serves a few purposes. First, a journal’s impact factor provides a baseline estimate for the expected number of citations an article will receive in a year. This isn’t a precise estimate; within a journal of a given impact factor, the citations of its articles can vary widely. As Fig 3 shows, despite the skew of citation distributions, high impact factors are not driven by outliers. Second, using impact factor as a matching covariate should help to account for some self-selection of authors and articles. Authors typically want to publish their articles in more prestigious, higher-impact journals; the more prestigious and high-impact the journal, the more selective it can be about publishing articles it expects to be highly cited. In our sample, as seen in Table 8, impact factor is strongly correlated with citations, and explains about 25% of the variance in citations.

**Table 8:** Regression of citations against journal impact factors. (t-statistics in parentheses).

	Citations (log scale)
Impact Factor (log scale)	0.974
	(0.010)
Intercept	0.700
	(0.013)
Observations	31,216
R^2^	0.246

**Fig 4:**
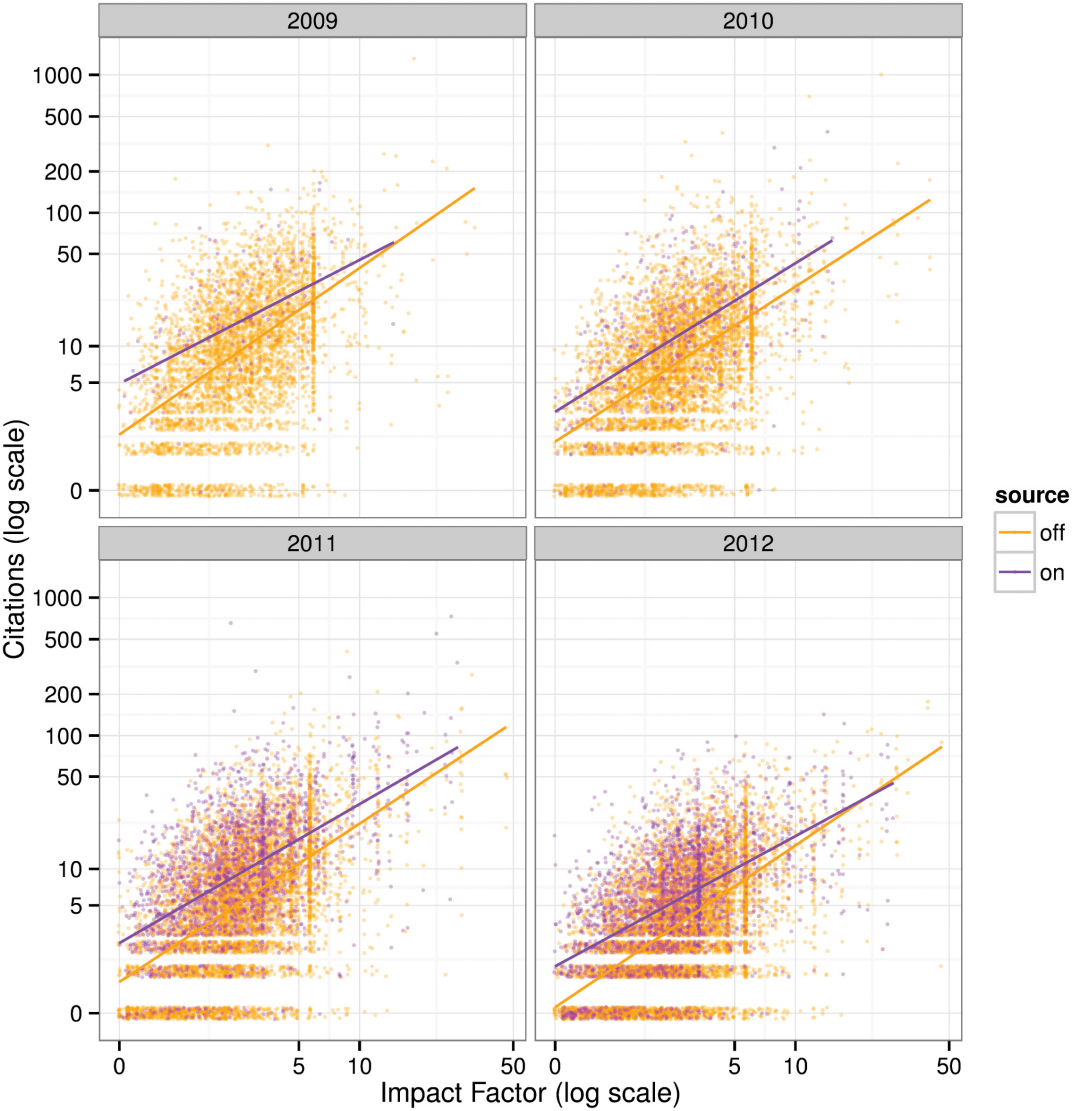
Citations against impact factors, with lines for conditional medians by off- and on-Academia sources.

Similar results can be seen in Fig 4, which shows scatter plots of article citations against journal impact factors (both on a log scale). The lines in the figure are predictions from separate median regressions for the on- and off-Academia group. Here we see the same result: a consistent citation advantage for on-Academia articles across cohorts and impact factors.

### Regression Analysis

We perform regression analyses with three different models:

1. A *linear regression* of log-scaled citation counts.
2. A *negative binomial regression* that explicitly models citations as (over-dispersed) counts.
3. A *zero-inflated negative binomial regression.* Motivated by the prevalence of uncited articles in our sample, we consider a mixture model of two negative binomial distributions. The first is a “zero” distribution that is degenerate at zero citations. Articles from this population will be uncited with probability one. The second is a “count” distribution, for articles that have a positive probability of being cited. The model estimates both the probability that an article belongs to the “zero” population and, conditional on coming from the “count” population, the probability distribution of an article’s citations given its features.

#### Covariates

We use the same covariates in all three regression models: (1) a dummy variable equal to one for articles posted to Academia.edu, (2) the article’s age (on a log scale) on the date its citation data was collected, (3) the impact factor of the article’s journal (on a log scale), (4) a dummy variable indicating whether the full-text of the article could be downloaded online from a non-Academia site, and (5) 23 dummy variables for the ANZSRC Divisions, indicating whether the article’s journal was labelled with each Division. Variables in (2)–(4) are interacted with the on-Academia dummy to allow for varying effects by age, impact factor, and online availability. In the linear and negative binomial models, the Division dummies in (5) are also interacted with the on-Academia dummy to obtain field-specific estimates of the on-Academia effect. These interactions were excluded from the zero-inflated negative binomial model as the model typically failed to converge when they were included.

Summary statistics for the age, impact factor, and online-availability variables are shown in Table 9.

**Table 9:** Summary statistics of regression model covariates.

	Mean	Median	Std. Dev.
**All**			
Age	3.07	2.89	1.06
Impact factor	2.92	2.30	2.77
Online	0.42	0.00	0.49
**On-Academia**			
Age	2.71	3.01	0.74
Impact factor	2.91	2.24	3.01
Online	0.67	1.00	0.47
**Off-Academia**			
Age	3.15	2.89	1.09
Impact factor	2.92	2.31	2.71
Online	0.37	0.00	0.48

#### Linear Regression

We fit a linear regression of log citations on the covariates described above. The coefficients on the on-Academia, year, online, and impact-factor covariates are listed in Table 10, in the column labelled “Linear.” For brevity, we exclude the 46 Division covariate and interaction coefficients. The age and impact factor coefficients have the expected signs and magnitudes. The coefficient of the on-Academia indicator is a statistically significant 0.433. Since the age and impact factor covariates are centered to have mean zero, the coefficient implies that for an article of the mean age and journal impact factor that is not available elsewhere online, posting to Academia.edu is associated with approximately 54% more citations.

**Table 10:** Regression results. Topic keyword coefficients omitted; t-statistics in parentheses.

	Linear	Neg. Binom.
(Intercept)	1.672 (102.478)	1.896 (102.287)
On-Academia	0.436 (10.480)	0.451 (9.699)
Impact factor (log, centered)	1.006 (88.985)	1.226 (94.539)
Article age (log centered)	0.834 (54.597)	1.086 (62.314)
Available online	0.201 (17.478)	0.219 (16.941)
On-Academia × Impact factor	-0.094 (-3.422)	-0.185 (-6.073)
On-Academia × Age	0.245 (5.059)	0.139 (2.591)
On-Academia × Available online	-0.054 (-1.887)	-0.032 (-0.985)
N	31216	31216
R-squared	0.348	
Deviance	23595.883	35125.570
Log-likelihood	-39925.553	-98494.268
AIC	79961.106	197098.536

The coefficient on “on-Academia × Online” is -0.054, indicating that for articles already freely available online, posting to Academia.edu is associated with a smaller difference in citations. At the sample average (see Table 9), the on-Academia coefficient for an online article is 0.436 − 0.054 = 0.38. The on-Academia × Age coefficient is positive, implying that the on-Academia effect increases with time.

The actual effect size of being on-Academia depends on the Division of the article, so is difficult to infer directly from the coefficient. We provide effect sizes based on typical values of the covariates in the next section.

#### Negative Binomial Regression

The negative binomial regression uses the same covariates as the linear regression, explicitly models citations as count data. The negative binomial distribution is a common choice for modeling over-dispersed count data.

In a negative binomial regression, the number of citations to article *i*, *y*_*i*_ is modeled as a function of covariates **x**_*i*_ according to:

$$y_{i}∼\text{NegBin}(\phi_{i},\theta )$$ (1)

$$\phi_{i}=e^{x_{i}\beta }$$ (2)

Fitting the regression provides estimates of the coefficients *β* and the scale parameter *θ*. Results for the entire sample are shown in Table 10 in the column “Neg. Binom.” We find similar results to the linear regression model—a large on-Academia coefficient that diminishes somewhat for articles available online elsewhere, but remains substantial. Again, due to the effects of Divisions, but also because the model is nonlinear, effect sizes are difficult to infer from the model coefficients, but we provide some effect size estimates in the next section.

#### Zero-Inflated Negative Binomial Regression

The modal number of citations for an article in our sample is zero or one, and approximately 11% of articles in our sample are uncited. Table 11 shows the share of uncited articles in each cohort of the off- and on-Academia samples. As expected, articles in newer cohorts are more likely to be uncited. But off-Academia articles are also much more likely to be uncited than on-Academia articles.

**Table 11:** Share of uncited articles in off- and on-Academia samples, by cohort.

Year	Off-Academia	On-Academia
2009	7.52%	1.34%
2010	8.77%	2.04%
2011	12.03%	4.03%
2012	16.89%	7.87%

To model these two aspects of the data, we fit a *zero-inflated* negative binomial model. This model assumes that an article comes from one of two populations: A “zero” population of articles that will be uncited with probability one, and a “count” population of articles whose citations will be drawn from negative binomial distributions conditioned on the articles’ features.

To represent the mixture of these two distributions, we add a second stage to the negative binomial model: a model of *z*_*i*_, which is equal to one when article *i* is from the “zero” distribution. *z*_*i*_ is assumed to come from a Bernoulli distribution that depends on the features of the article *x*_*i*_. We can write this as:

$$y_{i}∼\text{NegBin}(\phi_{i},\theta )$$ (3)

$$\phi_{i}=\left(1-z_{i}\right)e^{x_{i}\beta }$$ (4)

$$\left(1-z_{i}\right)∼\text{Bern}\left[{\text{logit}}^{-1}\left(x_{i}\gamma \right)\right]$$ (5)

When *z*_*i*_ = 1, then *ϕ*_*i*_ = 0, and the negative binomial distribution NegBin(0,*θ*) is degenerate at zero, and article *i* will have zero citations with probability one. Fitting the model estimates the *γ* and *β* coefficients. These are shown in Table 12.

**Table 12:** Coefficients from ZINB model. Division-on-Academia interactions were excluded to improve numeric stability.

	Count	Zero
(Intercept)	1.959 (114.361)	-6.295 (-14.239)
On-Academia	0.321 (12.244)	-7.041 (-2.102)
Impact factor (log, centered)	1.120 (79.201)	-5.436 (-15.772)
Article age (log centered)	1.052 (59.699)	-1.290 (-5.975)
Available online	0.217 (16.667)	-0.235 (-1.287)
On-Academia Impact factor	-0.112 (-3.981)	-3.935 (-1.316)
On-Academia Age	0.171 (3.194)	-4.966 (-1.147)
On-Academia Available online	-0.043 (-1.369)	-1.194 (-0.927)
Log(theta)	0.244 (24.274)	
N	31216	
Log-likelihood	-98208.100	
AIC	196542.200	

The coefficients in the “count” model are consistent with the linear and negative binomial regression coefficients in Table 10. In the “zero” model, though, we observe a large negative coefficient on the on-Academia dummy. This indicates, consistent with Table 11, that being posted on-Academia is associated with a much lower likelihood of being uncited. The on-Academia coefficient in the “count” model is smaller than the same coefficient in the Negative Binomial model. This implies that, compared only with off-Academia articles that have some positive probability of being cited at all, the on-Academia effect is somewhat smaller—though the lack of Division × on-Academia interactions in the zero-inflated model makes a direct comparisons difficult.

### Predicted Citation Advantages

Table 13 shows the predicted number of citations from the models above based on different values of the covariates. We predict citations for articles that:

**Table 13:** Predicted citations. Impact factor percentiles are based on the entire sample of articles. The Division variables are set to their sample means, which correspond to the share of articles labelled with that Division.

Model	IF Pctile	On-Academia	Online	1 Year	2 Years	3 Years	4 Years	5 Years
Linear	10th	N	N	0.14	1.03	1.84	2.61	3.35
			Y	0.39	1.48	2.47	3.42	4.32
		Y	N	0.32	1.79	3.33	4.90	6.51
			Y	0.53	2.24	4.01	5.84	7.70
	50th	N	N	1.31	3.12	4.78	6.35	7.85
			Y	1.83	4.04	6.06	7.98	9.82
		Y	N	1.52	4.32	7.24	10.24	13.30
			Y	1.92	5.16	8.54	12.02	15.56
	90th	N	N	3.71	7.39	10.77	13.95	17.01
			Y	4.76	9.26	13.38	17.28	21.02
		Y	N	3.80	9.14	14.70	20.41	26.24
			Y	4.56	10.74	17.19	23.81	30.56
NB	10th	N	N	0.91	1.93	3.00	4.11	5.23
			Y	1.13	2.41	3.74	5.11	6.52
		Y	N	1.27	2.96	4.86	6.92	9.10
			Y	1.53	3.57	5.87	8.35	10.98
	50th	N	N	2.16	4.59	7.13	9.75	12.42
			Y	2.69	5.72	8.88	12.14	15.47
		Y	N	2.64	6.17	10.13	14.42	18.95
			Y	3.18	7.44	12.23	17.40	22.87
	90th	N	N	5.14	10.92	16.96	23.18	29.54
			Y	6.41	13.60	21.12	28.87	36.79
		Y	N	5.50	12.86	21.14	30.08	39.54
			Y	6.64	15.52	25.51	36.29	47.71
ZINB	10th	N	N	0.81	1.95	3.13	4.33	5.55
			Y	1.05	2.48	3.95	5.44	6.94
		Y	N	0.69	3.05	5.06	7.19	9.45
			Y	1.29	3.66	6.02	8.56	11.25
	50th	N	N	2.32	4.83	7.41	10.04	12.70
			Y	2.89	6.00	9.21	12.47	15.77
		Y	N	2.68	6.28	10.31	14.65	19.25
			Y	3.20	7.47	12.27	17.44	22.91
	90th	N	N	5.16	10.69	16.39	22.18	28.05
			Y	6.40	13.28	20.35	27.55	34.84
		Y	N	5.48	12.80	21.02	29.88	39.26
			Y	6.52	15.23	25.02	35.57	46.73

1. Are in journals with impact factors at the 10th, 50th, or 90th percentiles of the sample;
2. Are one to five years old;
3. Are available online somewhere besides Academia.edu or are not;
4. Are either posted to Academia.edu or are not; and
5. Have values for the Division variables set to their sample means, i.e., the proportion of articles in the sample labelled with that Division.

The models give similar results, though the linear model tends to predict the lowest number of citations for any combination of covariates. Taking a three-year-old article published in a median impact factor journal as an example, the linear model predicts 4.78 citations for such articles not available on-Academia or elsewhere online, and 7.24 citations for such an article available only on Academia.edu—a difference of 2.5 citations or 51%. For a five-year-old article in a median impact factor journal, the linear model predicts 7.85 citations for a paper not available on Academia.edu or elsewhere online, and 13.30 citations for a paper available on Academia.edu—a difference of 5.5 citations, or 69%.

For articles available online elsewhere, but not on Academia.edu, the predicted number of citations after three years is 6.06. For articles available on Academia.edu and elsewhere online, the predicted number of citations is 8.54—a difference of 2.5 citations, or 41%. This number rises to 58% after five years (9.82 citations for articles available online elsewhere vs 15.56 for articles available on Academia.edu and elsewhere online).

Table 14 calculates the percentage increase in predicted citations, compared to an article not posted on Academia.edu and not available elsewhere online. If we measure the Academia.edu citation advantage as the percentage difference in citations to articles posted to on-Academia but not elsewhere online, then we find a range of estimates for the advantage depending on the age and impact factor, with the linear model predicting 16% in the first year to 69% in the fifth year for articles published in median impact factor journals. Consistent with the coefficients on the interaction term, the table shows that the advantage decreases for higher impact factor journals, which expect more citations just from being published. For example, we find that the Academia.edu citation advantage for a paper published in a high impact factor journal is 2% in the first year, rising to 37% in the third year, and 54% in the fifth.

**Table 14:** Predicted citation advantages relative to paid-access articles, from Table 13.

Model	IF Pctile	On-Academia	Online	1 Year	2 Years	3 Years	4 Years	5 Years
Linear	10th	N	N	—	—	—	—	—
			Y	1.84	0.44	0.34	0.31	0.29
		Y	N	1.35	0.75	0.81	0.88	0.94
			Y	2.88	1.18	1.18	1.23	1.30
	50th	N	N	—	—	—	—	—
			Y	0.39	0.29	0.27	0.26	0.25
		Y	N	0.16	0.38	0.51	0.61	0.69
			Y	0.46	0.65	0.79	0.89	0.98
	90th	N	N	—	—	—	—	—
			Y	0.28	0.25	0.24	0.24	0.24
		Y	N	0.02	0.24	0.37	0.46	0.54
			Y	0.23	0.45	0.60	0.71	0.80
NB	10th	N	N	—	—	—	—	—
			Y	0.25	0.25	0.25	0.25	0.25
		Y	N	0.39	0.53	0.62	0.69	0.74
			Y	0.68	0.85	0.95	1.03	1.10
	50th	N	N	—	—	—	—	—
			Y	0.25	0.25	0.25	0.25	0.25
		Y	N	0.22	0.34	0.42	0.48	0.53
			Y	0.47	0.62	0.71	0.78	0.84
	90th	N	N	—	—	—	—	—
			Y	0.25	0.25	0.25	0.25	0.25
		Y	N	0.07	0.18	0.25	0.30	0.34
			Y	0.29	0.42	0.50	0.57	0.61
ZINB	10th	N	N	—	—	—	—	—
			Y	0.31	0.27	0.26	0.26	0.25
		Y	N	-0.14	0.56	0.62	0.66	0.70
			Y	0.60	0.88	0.92	0.98	1.03
	50th	N	N	—	—	—	—	—
			Y	0.24	0.24	0.24	0.24	0.24
		Y	N	0.16	0.30	0.39	0.46	0.52
			Y	0.38	0.55	0.65	0.74	0.80
	90th	N	N	—	—	—	—	—
			Y	0.24	0.24	0.24	0.24	0.24
		Y	N	0.06	0.20	0.28	0.35	0.40
			Y	0.27	0.42	0.53	0.60	0.67

The second row of each model/impact-factor panel in Table 14 gives an advantage estimate for article available online but not on Academia.edu. These are estimates of the general Open Access advantage in our data, and are about 25% for three year-old articles.

While Tables 13 and 14 provide point estimates for predicted citations under a variety of scenarios, Table 15 shows the confidence and prediction intervals around the predicted citations for two example scenarios: three- and five-year old articles from a median impact factor journal, estimated by the linear model. As expected given the statistically significant coefficient estimates, the confidence intervals around predictions for different online and on-Academia states are relatively narrow and show statistically significantly different predictions. The prediction intervals are relatively wide, driven by the remaining unexplained variance in the model (*R*^2^ = 0.35, RMSE = 0.87 log citations).

**Table 15:** Predicted citations from the Linear model, with confidence and prediction intervals.

Age	On-Academia	Online	Pred. Cites	95% Conf. Int.	95% Pred. Int.
3	N	N	4.78	(4.7, 4.86)	(0.05, 30.81)
		Y	6.06	(5.94, 6.19)	(0.28, 37.88)
	Y	N	7.24	(6.85, 7.64)	(0.5, 44.34)
		Y	8.54	(8.2, 8.9)	(0.73, 51.52)
5	N	N	7.85	(7.66, 8.03)	(0.61, 47.7)
		Y	9.82	(9.56, 10.08)	(0.96, 58.53)
	Y	N	13.31	(12.25, 14.42)	(1.6, 77.68)
		Y	15.57	(14.48, 16.72)	(2.01, 90.15)

#### Citation Advantages by Division

In Table 16, we predict the citation advantage for three-year old articles published in the median impact factor journal within each Division. The advantage estimates range from 55% to 160%, with the largest estimates coming from Divisions with lower median impact factors.

**Table 16:** Predicted citations and on-Academia citation advantages by Division for five year old articles. Citations are predicted from the “Linear” model in Table 10, and are calculated for five year old articles from journals with the median impact factor of the Division. Articles are assumed to have a single Division.

Division	Med. IF	% On	% Off	Cites Off	Cites On	Diff.	% Adv.
History & Archaeology	0.92	4.8%	0.5%	2.58	6.71	4.13	160%
Education	1.12	4.5%	1.2%	5.33	12.12	6.79	127%
Creative Arts & Writing	0.76	1.4%	0.3%	3.82	7.98	4.15	109%
Law and Legal Studies	0.77	0.8%	0.3%	3.69	7.41	3.72	101%
Info. & Comp. Sciences	1.95	5.2%	4.8%	6.58	13.05	6.47	98%
Lang., Communcn. & Culture	0.63	4.6%	0.8%	3.51	6.88	3.37	96%
Commerce, Mgt., Tourism & Svcs.	1.30	4.4%	2.5%	6.60	12.82	6.22	94%
Earth Sciences	1.96	1.9%	2.3%	6.14	11.79	5.64	92%
Technology	2.28	8.7%	2.9%	7.80	14.95	7.16	92%
Agricultural & Vet. Sciences	2.16	4.6%	3.5%	7.92	15.13	7.22	91%
Psych. & Cognitive Sciences	2.46	17.5%	5.6%	8.42	15.86	7.44	88%
Physical Sciences	2.41	8.3%	7.0%	6.81	12.54	5.73	84%
Mathematical Sciences	1.36	5.0%	7.5%	5.26	9.55	4.29	81%
Economics	1.15	1.9%	1.5%	6.00	10.80	4.80	80%
Philosophy & Religious Studies	0.64	4.2%	0.8%	3.59	6.27	2.68	75%
Engineering	2.77	12.0%	25.3%	8.83	15.21	6.38	72%
Studies in Human Society	1.15	9.8%	2.4%	5.53	9.49	3.97	72%
Medical & Health Sciences	2.58	18.6%	36.1%	8.25	14.09	5.84	71%
Environmental Sciences	2.48	5.3%	3.0%	8.06	13.64	5.58	69%
Biological Sciences	2.55	19.6%	20.8%	8.36	13.94	5.58	67%
Chemical Sciences	3.79	6.3%	21.4%	11.03	18.00	6.97	63%
Multidisciplinary	3.20	11.5%	3.7%	10.53	17.13	6.59	63%
Built Environment & Design	1.84	1.7%	0.8%	6.79	10.56	3.77	56%

## Discussion

### Issues and Topics for Further Research

Our results raise several questions that warrant further research. One area to consider is which properties of Academia.edu are responsible for papers receiving more citations. We observed that the Academia.edu citation advantage is distinct from a general open access advantage; even amongst papers posted online elsewhere, those that are also posted on Academia.edu receive more citations. One hypothesis is that Academia.edu goes to various lengths to expose posted paper to other users. Academia.edu users are actively notified about papers posted by users they follow and in research topics they follow. This may provide more articles with more exposure than they otherwise would have had, which may lead to more citations. Further work could be done to measure the effect of these distributional properties.

Another line of study relates to the dynamics of citations. In this study, we have looked at citation counts at a fixed moment in time. Other studies, notably [19], have looked at the accumulation of citations over time. Having longitudinal data on citations would help us answer several questions. For articles uploaded to Academia.edu after they were published—which we exclude from this study—we could test for a change in the rate of citations received after uploading. For articles posted at the same time they’re published—which we did study here—we could analyse to what extent there are feedback effects. Is the relatively large citation advantage a result of being more likely to receive the first one or two citations from posting to the site?

Beyond Academia.edu, our work raises questions about how characteristics of venues matter for open access citations. To our knowledge there has been no research on what features of open access repositories or databases make articles easier to discover, and to what extent that leads to increased citations.

### Conclusions

We have analyzed the effect of open access on citations using a novel venue for free-to-access articles, Academia.edu. Using a matching analysis and regression models with covariates to control for potential sources of selection bias, we find a substantial increase in citations associated with posting an article to Academia.edu. We find that a typical article posted to Academia.edu has 69% more citations than a similar paid-access article, not available elsewhere online, after five years. We find that a typical article that is also posted to Academia.edu has 49% more citations than one that is only available elsewhere online through a non-Academia.edu venue: a personal or departmental homepage, a journal site, a repository such as ArXiV or SSRN, or any other online hosting venue.

While the true effect of open access on citations remains debated in the literature, the effect we find here suggests that features that improve the discoverability, such as the feeds and notifications used on Academia.edu, may be important factors in determining how much open access increases citations. We believe more research along these lines would help improve our understanding of the causal mechanisms behind the open access citation advantage, help researchers make better decisions about how to provide access to their research, and help journals and institutions make their open access policies more effective.
